# The global trends and hotspots beta-blocker therapy in sepsis and septic shock: A bibliometric analysis based on CiteSpace and VOSviewer

**DOI:** 10.1097/MD.0000000000048347

**Published:** 2026-04-24

**Authors:** Xu-Ying Luo, Jianfang Zhou, Jie Zheng, Guangzhi Shi, Hong-Liang Li

**Affiliations:** a Department of Critical Care Medicine, Beijing Tiantan Hospital, Capital Medical University, Beijing, China; b Jie Zheng Consulting Limited, Bolton, United Kingdom; c Department of Critical Care Medicine, Beijing Shijitan Hospital, Capital Medical University, Beijing, China; d Emergency and Critical Care Medical Center, Beijing Shijitan Hospital, Capital Medical University, Beijing, China.

**Keywords:** bibliometric study, research trends, sepsis, septic shock, β-blocker

## Abstract

Beta-blocker therapy has garnered attention for sepsis and septic shock because of its potential benefits in modulating hemodynamics through heart rate control and reducing the in-hospital mortality rate. This study examined the evolving trends and focal points of this domain. This study retrieved 418 articles and reviews related to beta-blocker therapy in sepsis and septic shock published from the Web of Science Core Collection Database from January 1, 1990, to November 30, 2023. CiteSpace and VOSviewer software were used for visual bibliometric analysis, including co-citation analysis of references, co-occurrence analysis of keywords, and collaborative network analysis involving countries/regions, institutions, and authors. Over time, a discernible upward trend has been observed in the annual publication of articles related to β-blockers in sepsis, particularly from 2013 to 2023. Keyword publication burst detection revealed that initial studies primarily focused on the role of excessive catecholamine release as a mechanism and potential therapeutic target in sepsis and septic shock. β-blockers have emerged as a pivotal area of investigation and are associated with improved hemodynamics and decreased in-hospital mortality. The United States is the most prolific contributor, wielding substantial influence, whereas China exhibits a recent surge in publications, ranking second in terms of publication strength. University College London published the most articles, with the University of Oxford demonstrating the highest centrality. Collaborative endeavors are frequently observed among countries and institutions. Beta-blocker therapy may hold promise in reducing in-hospital mortality among patients with sepsis and septic shock, thus becoming a central focus of research.

## 1. Introduction

Sepsis, defined by the Sepsis-3 criteria, is a life-threatening condition characterized by organ dysfunction, resulting from a dysregulated host inflammatory response to infection.^[1]^ Despite advances in therapeutic strategies, in-hospital mortality associated with sepsis remains alarmingly high, especially in cases accompanied by shock.^[2,3]^ The mechanisms of sepsis and septic shock are intricate, involving activation of the sympathetic nervous system, which leads to excessive catecholamine release and increased cytokine production, compromised cardiovascular and immune function, and hypermetabolism.^[4–7]^ In this context, β-blockers, well established for their use in coronary artery disease and chronic heart failure, have emerged as a potential therapeutic option for improving sepsis outcomes. A growing body of evidence suggested that β-blockers may protect against cardiac dysfunction by controlling the heart rate and were associated with decreased mortality.^[8–10]^ The precise mechanisms underlying this beneficial effect remain elusive, potentially involving antisympathetic and immunomodulatory.^[10,11]^ However, β-blocker administration may cause hypotension and decrease systolic function, especially in patients with compensated tachycardia. Therefore, the optimal timing and dosage of β-blocker administration in patients with sepsis and septic shock remain a topic of debate.

Bibliometrics, a collection of mathematical and statistical methods, offers quantitative insights into literature. They help estimate research output and collaborations among researchers, institutions, and countries, and identify research trends using cluster and burst analyses of keywords and references within a specific research domain.^[12]^ In this study, we employed bibliometric analysis using CiteSpace and VOSviewer software to examine publication patterns, explore developments, and identify trends in beta-blocker therapy for sepsis and septic shock.

## 2. Materials and methods

### 2.1. Data source and search strategy

To gather relevant literature on “beta-blocker therapy in sepsis,” we searched the Web of Science (WOS) Core Collection database from January 1, 1990 to November 30, 2023. A literature search was performed on December 1, 2023. We focused on original research and review articles written in English.

The search strategy included the following terms: TS = (“beta-blocker^*^” OR “beta-blockade^*^” OR “beta-adrenergic blocker^*^” OR “adrenergic beta antagonist^*^” OR “adrenergic beta-1 receptor antagonist^*^” OR “adrenergic beta-2 receptor antagonist^*^” OR “landiolol” OR “esmolol^*^” OR “propranolol^*^” OR “bisoprolol^*^” OR “metoprolol^*^”) AND TS = (sepsis OR “septic shock” OR “severe sepsis”). This strategy yielded 418 bibliographic records, original articles, and reviews in English. Duplicate records were meticulously screened, and none were found.

### 2.2. Bibliometric analysis

We utilized CiteSpace 6.1.R3 and VOSviewer 1.6.20 for our bibliometric analysis, enabling a comprehensive field examination. CiteSpace was developed by Chaomei Chen at Drexel University (Philadelphia),^[13]^ and VOSviewer was developed by the Centre for Science and Technology Studies (CWTS) at Leiden University (Leiden, Netherlands).^[14]^ Our analysis included a co-citation analysis of references to elucidate the research foundation of this domain. Furthermore, we employed keyword co-occurrence, cluster analysis, and burst detection to uncover the prevailing research trends and hotspots. Finally, we conducted a co-occurrence analysis of researchers, institutions, and countries to delineate global research collaborations. Our study has no direct connection to specific patients. Thus, an ethical evaluation is not required.

## 3. Results

### 3.1. Annual publication and trends

**Figure 1.**
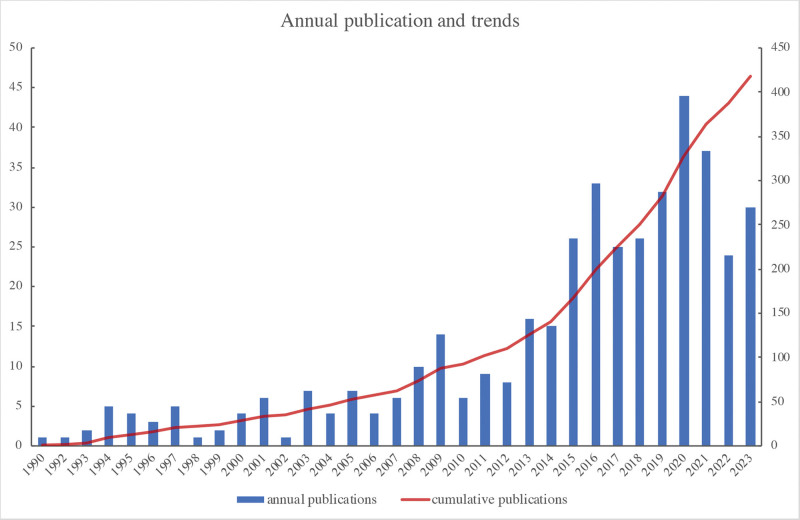
Annual and cumulative publication counts.

A total of 418 publications, comprising 313 articles and 88 reviews, were sourced from the WOS Core Collection database for the analysis. Figure 1 illustrates the annual and cumulative number of publications. The data revealed a discernible upward tendency in annual publications from 1990 to 2023. This growth can be divided into 2 distinct phases are as follows: an initial period from 1990 to 2012, characterized by steady but slow growth, followed by a substantial surge from 2013 to 2023, which signifies a noteworthy global escalation in interest regarding the potential protective properties of β-blockers in sepsis and septic shock.

### 3.2. Keywords co-occurrence analysis

**Figure 2.**
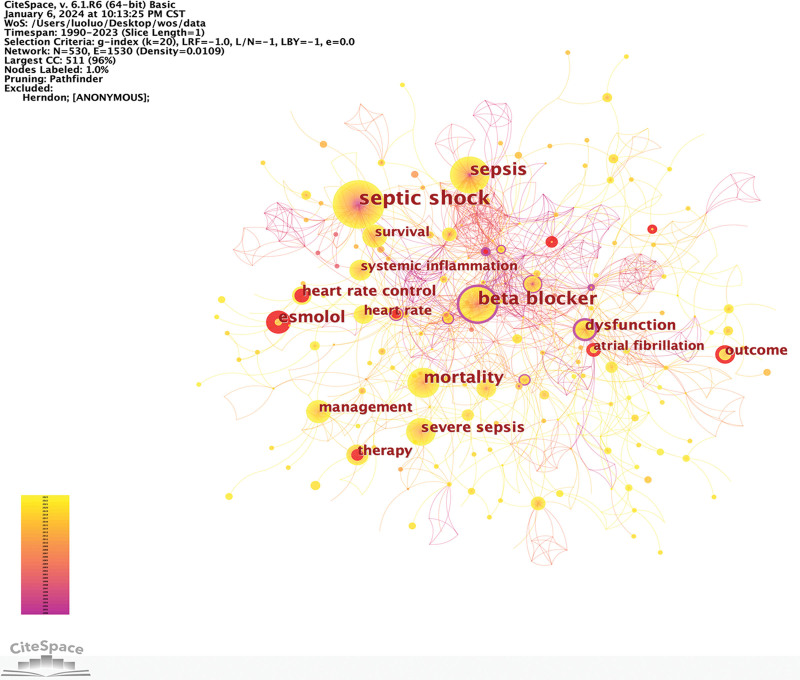
Co-occurrence network of keywords. CC = co-occurrence, LBY = look back years, LRF = link retaining factor, WOS = Web of Science.

Keyword co-occurrence analysis is used to pinpoint research hotspots. To ensure comprehensive coverage, amalgamated various terms such as “beta blocker,” “beta-blockade,” “beta-adrenergic blocker,” “adrenergic beta-antagonists,” and “adrenergic beta-1 receptor antagonists.” From 1990 to 2023, the resulting keyword network contained 530 nodes and 1530 links, as shown in Figure 2. Notably, the top 5 keywords in terms of co-occurrence are “septic shock,” “beta blocker,” “sepsis,” “mortality,” and “esmolol,” signifying the primary research focal points.

**Figure 3.**
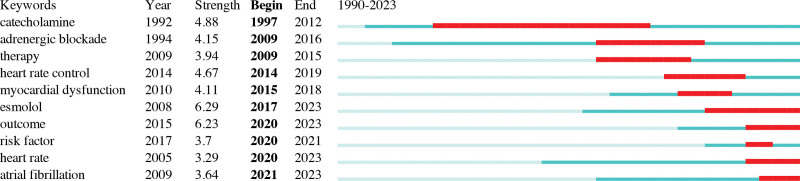
Top 10 keywords with publication bursts sorted by start time.

In this network, “catecholamine” emerged with the highest centrality (0.38), which underscores the pivotal role of excess catecholamine release as a critical pathophysiological mechanism in the early phase of sepsis, subsequently leading to the exploration of β-blockers as a potential therapeutic avenue. Figure 3 shows the top 10 keywords exhibiting publication bursts arranged chronologically. The earliest surge pertains to “catecholamine” (1997–2012), followed by “adrenergic blockade” (2009–2016), and “therapy” (2009–2015). These early investigations primarily delved into the phenomenon of excess catecholamine release in sepsis and then shifted toward therapeutic strategies.

In recent years, the emergence of keywords such as “esmolol” (2017–2023), “outcome” (2020–2023), “heart rate” (2020–2023), and “atrial fibrillation” (2021–2023) indicates a contemporary research focus. It is worth noting that new-onset atrial fibrillation, occurring in approximately 20% of sepsis patients, has garnered significant attention due to its association with increased stroke and mortality risks.^[15,16]^ Potential mechanisms involve excessive catecholamine release, systemic inflammation, autonomic dysfunction, and ischemia, all of which contribute to the pathogenesis of atrial fibrillation.^[17]^ Atrial fibrillation, characterized by rapid heart rates and diminished cardiac output, exacerbates hemodynamic instability in sepsis and septic shock, thus explaining the heightened research interest.^[18]^

Among the top 5 keywords exhibiting the most robust burst strength, “esmolol” (6.29), “outcome” (6.23), “catecholamine” (4.88), “heart rate control” (4.67), and “adrenergic blockade” (4.15) underscore their paramount significance within this field. Notably, “esmolol” stands out because of its sustained burst duration, which extends until 2023, signifying evolving research directions.

### 3.3. References co-citation analysis

**Figure 4.**
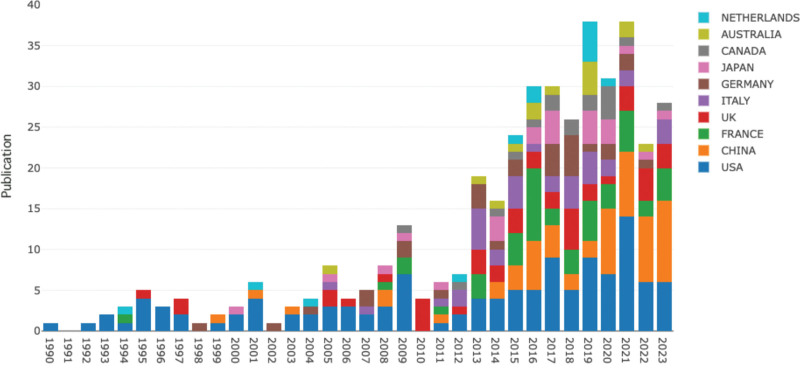
The annual publication by countries/regions from 1990 to 2023.

Figure 4 shows the reference co-citation network generated from publications between 1990 and 2023, constructed using the top 50 most-cited publications per year, and it contained 846 nodes and 2129 links. Its high modularity score of 0.9392 reflects well-defined co-citation clusters in scientific mapping, and an average silhouette score of 0.9538 indicates the reliability of the clustering results.

Table 1 presents the top 10 most significant reference clusters, with Cluster #0 focusing on heart rate control emerging as the largest, comprising 62 articles with an average publication year of 2010. A pivotal study by Aboab et al demonstrated that continuous esmolol infusion, titrated to reduce heart rate by 20%, counteracted lipopolysaccharide-induced reductions in cardiac and stroke indices in septic pigs.^[19]^ Similarly, Mori et al reported that esmolol improved the mean survival time in septic rats, potentially by modulating gut mucosal integrity and local inflammatory response.^[20]^ Moreover, long-term β-blocker use has demonstrated protective effects on the cardiovascular system in sepsis. Ackland et al reported that metoprolol exerted anti-inflammatory and cardioprotective effects, reducing mortality in rats subjected to septic insults.^[10]^ Subsequently, a large-scale retrospective study involving 9465 septic intensive care unit (ICU) patients in Italy validated the association between preexisting β-blocker use and reduced 28-day mortality.^[21]^

**Table 1:** Top 10 largest references co-citation clusters.

Rank	Cluster ID	Size	Silhouette	Mean year	Label (LLR)
1	0	62	0.957	2010	Heart rate control
2	1	58	0.89	2018	Preexisting heart failure
3	2	58	0.949	2014	Vascular function
4	3	55	0.985	2006	Adrenergic modulation
5	4	41	0.9	2016	Vasopressor therapy
6	5	38	0.99	2012	Proton pump inhibitor
7	6	33	0.966	2017	Narrative review
8	12	18	0.974	2016	Sepsis-related tachyarrhythmia
9	13	16	0.999	2019	EASL clinical practice guideline
10	18	13	0.998	2018	New-onset atrial fibrillation

EASL = European Association for the Study of the Liver, LLR = log-likelihood ratio.

Cluster #1 is the second largest cluster, containing 58 articles published from 2013 to 2022, with an average publication year of 2018. This cluster affirms the protective effects of β-blockers through meta-analyses. A meta-analysis incorporating 5 multicenter randomized controlled trials (RCTs) demonstrated that esmolol reduced heart rate and cardiac troponin I levels while potentially improving survival without adversely affecting hemodynamic parameters in patients with sepsis and septic shock.^[22]^ In addition, another meta-analysis incorporating 7 RCTs revealed that ultrashort-acting β-blockers, such as esmolol and landiolol, were associated with lower 28-day mortality in septic patients with persistent tachycardia despite adequate resuscitation.^[9]^ Furthermore, Fuchs et al ascertained that continued long-term beta-blocker therapy during the acute phase of sepsis and septic shock was linked to decreased 90-day mortality.^[23]^ However, the optimal timing for administering β-blockers remains a topic of contention, despite extensive preclinical and clinical data supporting their safety.^[24]^

Cluster #2 centers on vascular function and contains 58 articles with an average publication year of 2014. This cluster focused on the intricacies of vascular function in patients with septic shock. Notable contributions include Jacquet-Lagrèze et al, who reported that esmolol improved the gut and sublingual microcirculatory blood flow during septic shock in a porcine model.^[25]^ Similarly, Kimmoun et al demonstrated that esmolol enhanced intrinsic cardiac contractility and vascular responsiveness to catecholamines in rats with septic shock.^[26]^ A prospective study involving 45 septic shock patients with persistent tachycardia after at least 24 hours of hemodynamic stabilization revealed that esmolol improved cardiac and arterial elastance while reducing norepinephrine dosage, without compromising cardiac output and ejection fraction.^[27]^ Moreover, 6 articles within this cluster are among the top 10 most-cited publications.^[8,26–30]^

The top 10 most-cited publications are presented in Table S1, Supplemental Digital Content, https://links.lww.com/MD/R663. Notably, the article “Effect of Heart Rate Control with Esmolol on Hemodynamic and Clinical Outcomes in Patients with Septic Shock: A Randomized Clinical Trial” was a seminal work.^[8]^ This is the first randomized clinical trial to investigate the effects of esmolol, a short-acting β-blocker, on heart rate reduction and mortality in patients with septic shock without a concomitant increase in adverse events. This study also obtained the highest citation burst score (25.09). Five of the top 10 articles explored the use of β-blockers in preserving microvascular blood flow, enhancing cardiovascular function, and potential mechanisms in sepsis and septic shock.^[10,19,26–28]^ In addition, among the top 10 most-cited publications were 2 practice guidelines: “Surviving Sepsis Campaign: International Guidelines for Management of Severe Sepsis and Septic Shock, 2012,” which underscored the importance of early goal-directed therapy, including 3- and 6-hour bundles,^[29]^ and “The Third International Consensus Definitions for Sepsis and Septic Shock (Sepsis-3),” which proposed a novel definition of sepsis as “a life-threatening organ dysfunction caused by a dysregulated host response to infection.”^[1]^ These updated definitions enhance clinical management and ensure greater consistency in epidemiological studies and clinical trials.

**Figure 5.**
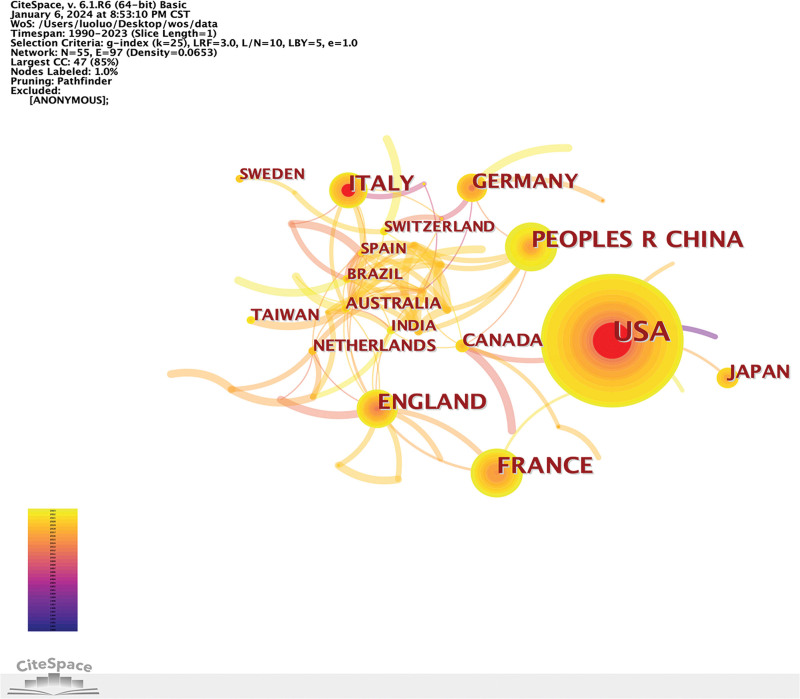
Collaboration network among countries/regions. CC = co-occurrence, LBY = look back years, LRF = link retaining factor, WOS = Web of Science.

Figure 5 illustrates the top 10 references that exhibited the most significant citation bursts sorted by their inception dates. The first 2 publications underscored the potential anti-inflammatory and cardioprotective effects of β1-blockers and decreased mortality in animal experiments.^[10,19]^ Subsequently, researchers have explored the underlying mechanisms responsible for the cardiovascular effects of esmolol. Notably, Morelli et al demonstrated that titrated esmolol infusion, aimed at reducing heart rates below 95 beats/min, was associated with preserved sublingual microvascular blood flow, reduced norepinephrine requirements, decreased 28-day mortality, and improvements in other secondary clinical outcomes among septic shock patients.^[28]^ Esmolol improves intrinsic cardiac contractility and vascular responsiveness to catecholamines, primarily because of its anti-inflammatory effects.^[26]^ Furthermore, a multicenter RCT revealed that ultrashort-acting β1-blockers reduced the incidence of new-onset arrhythmias and improved survival.^[31]^ These findings were corroborated by a systematic review and meta-analysis.^[9]^

### 3.4. Publication by countries/regions

**Figure 6.**
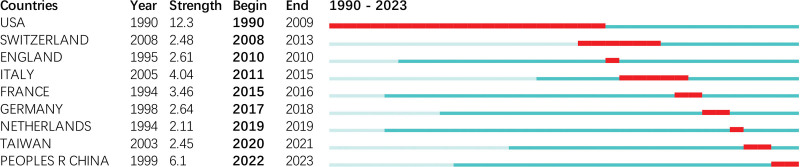
Publication bursts of countries/regions sorted by start time.

A total of 55 countries/regions contributed to relevant publications, with the United States leading in article production (120), followed by France (46), China (46), England (40), and Italy (34). Figure 4 illustrates the annual publications by countries/regions from 2005 to 2023. Moreover, complex international collaborations were evident (see Fig. 5). The United States exhibits the highest centrality score (0.47), followed by England (0.24) and Italy (0.2), indicating their essential contributions to this field. Analyzing publication bursts by inception date (Fig. 6), the United States had the earliest, most prolonged, and most substantial bursts from 1990 to 2009. Conversely, China recently experienced a publication burst in 2022, ranking second in strength (6.1).

### 3.5. Analysis of institutions and authors

**Figure 7.**
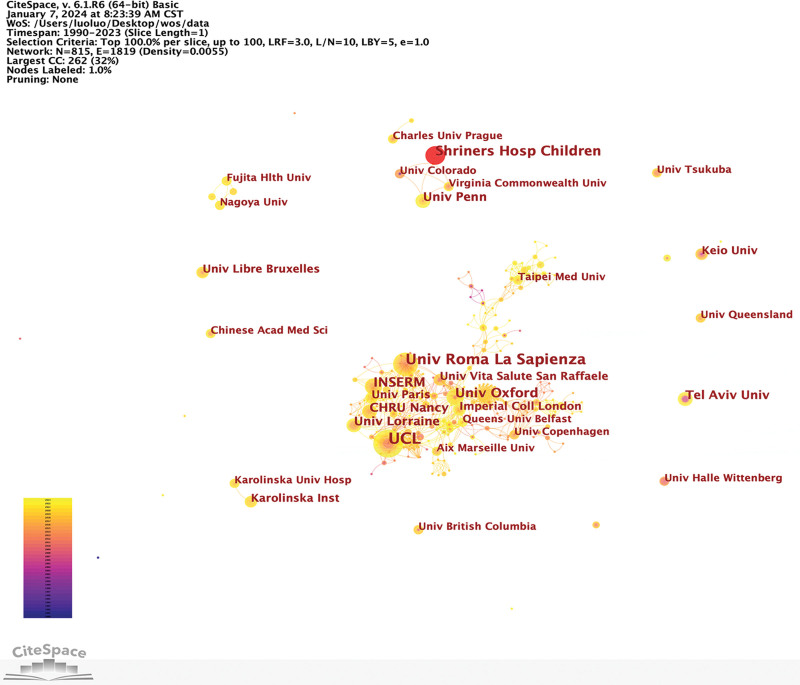
Collaboration network among institutions. CC = co-occurrence, LBY = look back years, LRF = link retaining factor, WOS = Web of Science.

Eight hundred fifteen institutions have contributed relevant articles, often engaging in collaborative efforts, particularly within the same country (Fig. 7). The top 10 most productive institutions are listed in Table 2, with University College London leading the list (12 publications), followed by Sapienza University of Rome (10), Shriners Hospitals for Children (8), The National Institute of Health and Medical Research (INSERM) (7), and the University of Oxford (7). Among the top 10 institutions, 3 were in the United States, 3 in France, and 2 in England. Shriners Hospitals for Children and the University of Texas were the earliest to burst onto the scene, initiating their research in 2001 with the most substantial burst strength (3.92 and 3.22, respectively). Shriners Hospitals for Children maintained the most extended publication burst from 2001 to 2014, concentrating on the impact of propranolol as a metabolic modulator in sepsis, especially in severely burned children.^[32–34]^ Nagoya University and Fujita Health University in Japan recently exhibited publication bursts from 2020 to 2021 (Fig. S1, Supplemental Digital Content, https://links.lww.com/MD/R664). The University of Oxford boasted the highest centrality score (0.1) and provided comprehensive summaries of β-blocker use in sepsis and new-onset atrial fibrillation among ICU patients, thus bridging critical knowledge gaps.^[16,30,35,36]^

**Table 2:** Top 10 most productive institutions.

Rank	Frequency	Centrality	Year	Institution	Country
1	12	0.08	2010	UCL	England
2	10	0.06	2013	Univ Roma La Sapienza	Italy
3	8	0.01	2001	Shriners Hosp Children	USA
4	7	0.01	2015	INSERM	France
4	7	0.1	2012	Univ Oxford	England
6	6	0	2007	Tel Aviv Univ	Israel
6	6	0.01	2016	CHRU Nancy	France
6	6	0.01	2015	Univ Lorraine	France
6	6	0.09	2015	Univ Penn	USA
10	5	0	2001	Univ Texas	USA
10	5	0	2005	Keio Univ	Japan
10	5	0	2018	Karolinska Inst	Sweden
10	5	0.02	2017	Univ Paris	France
10	5	0.03	2014	Univ Libre Bruxelles	Belgium
10	5	0.04	2017	Imperial Coll London	England
10	5	0.06	2012	Univ Vita Salute San Raffaele	Italy

UCL = University College London.

**Figure 8.**
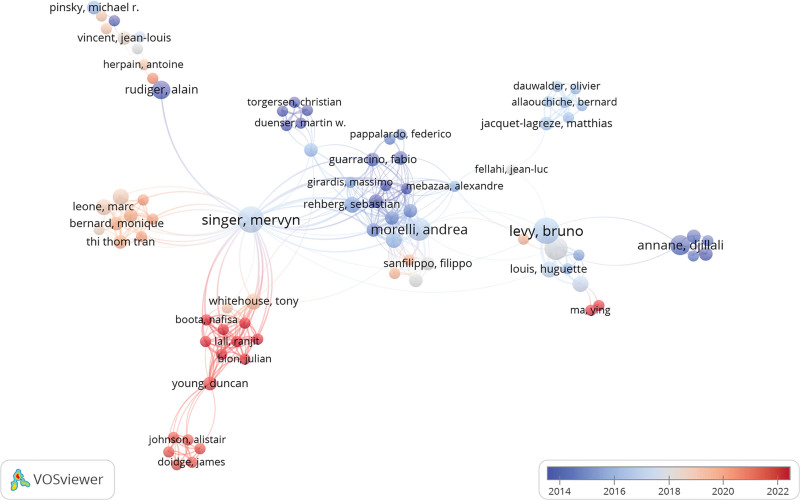
Collaboration network among authors.

**Figure 9.**
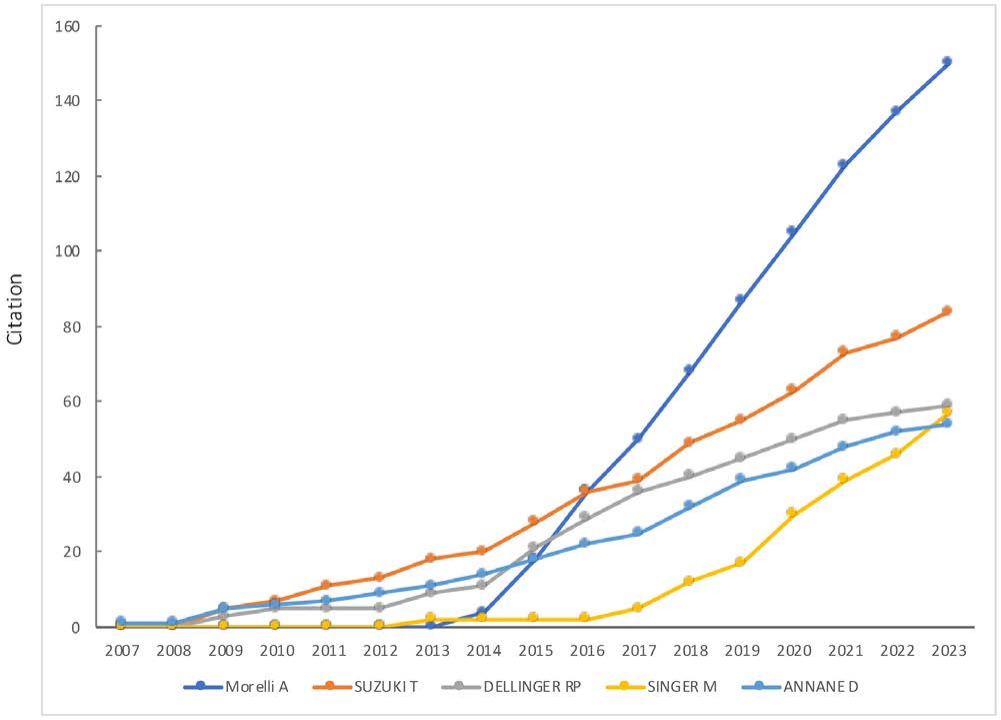
Cumulative citation distribution of the top 5 most-cited authors by year.

Two thousand three hundred eleven authors contributed to pertinent articles, often collaborating, especially those with numerous publications (Fig. 8). Table 3 presents the top 10 most productive authors and the top 10 authors with the highest citations. Notably, the total citations of the top 5 most-cited authors have sharply risen from 2013 to 2023 (Fig. 9), reflecting the growing prominence of β-blockers in sepsis and septic shock research. Authors such as Singer, Morelli, Annane, Herndon, and Rudiger have earned positions in both the most cited and most productive categories, underscoring their substantial contributions to this field.

**Table 3:** Top 10 most productive authors and the top 10 authors with the highest citations.

Rank	Count	Centrality	Authors	Co-cited authors	Count	Centrality
1	10	0.03	Singer	Morelli	150	0
2	10	0.01	Levy	Suzuki	84	0.01
3	8	0	Kimmoun	Dellinger	59	0.05
4	8	0	Morelli	Singer	57	0.01
5	6	0	Annane	Annane	54	0.32
6	6	0	Herndon	Ackland	52	0.2
7	5	0.01	Rudiger	Rudiger	51	0.07
8	5	0	Matthay	Schmittinger	46	0.01
9	4	0	Van Der Hoeven	Vincent	44	0.05
10	4	0	Jeschke, Marc	Herndon	44	0.38

Singer and Levy hold the distinction of having the most publications (Table 3). Singer published the pivotal Sepsis-3, a milestone in sepsis research, and acting as a collaborator author, focusing on the effects of ultrashort-acting β-blockers such as esmolol and landiolol in sepsis and septic shock, including cardiovascular system protection, immunoregulation, sex differences, and potential mechanisms.^[10,36–38]^ Morelli from the Sapienza University of Rome ranked fourth in the list of the top 10 most publications, with 3 articles among the top 10 most-cited publications. As the first author, Morelli contributed 4 articles primarily focusing on the effect of heart rate control using esmolol on the cardiovascular system and clinical outcomes in patients with septic shock.^[8,27,28,39]^ His work was first cited in 2014, surpassing others, and ranked first in 2017.

## 4. Discussion

Bibliometric analysis of recent literature has demonstrated that the protective effects of β-blockers in sepsis and septic shock have become a research hotspot over the past decade. However, controversies remain regarding the specific septic phenotypes that might derive benefits from β-blocker administration. Thus, elucidating the underlying pathophysiological mechanisms and identifying subgroups of patients that could benefit from β-blocker use may represent a promising area for future research.

Sepsis is characterized by a dysregulated host inflammatory response to infection and frequently involves organ dysfunction and high in-hospital mortality.^[1]^ Sympathetic overstimulation, characterized by persistent tachycardia, played a pivotal role in the acute phase of sepsis.^[4,5,7]^ β-blockers, primarily used for coronary artery disease and chronic heart failure, have garnered significant attention owing to their potential to reduce mortality in sepsis and septic shock.^[10,40]^ Keyword publication burst detection has traced research development. Keywords such as septic shock, β-blocker, sepsis, mortality, and esmolol topped the list, underscoring the central focus of research in this field. Early studies of adrenergic blockade in sepsis focused on pathophysiological mechanisms, including amelioration of sympathetic overactivity, attenuation of hypermetabolism, and regulation of immune responses,^[10,19,20]^ and burst for clinical application from 2009 to 2016. A landmark randomized clinical study demonstrated that esmolol was associated with a reduced heart rate and decreased mortality without increasing adverse events.^[8]^ Subsequent publication bursts related to heart rate control (2014–2019), myocardial dysfunction (2015–2018), and esmolol (2017–2023) emerged. The most recent publication bursts focus on outcomes (2020–2023), heart rate (2020–2023), and atrial fibrillation (2021–2023) since new-onset atrial fibrillation is a common occurrence in sepsis, compromising hemodynamics and correlating with increased morbidity and mortality.^[41]^

Although short-acting β-blockers have demonstrated potential protective effects in patients with sepsis and septic shock,^[9,22,31]^ conflicting results have also been reported. The negative chronotropic and inotropic effects of β-blockers may lead to hypotension and decreased cardiac index.^[42]^ Consequently, the therapeutic efficacy of short-acting β-blockers in septic shock may depend on the patient’s hemodynamic status, including preload and cardiac systolic and diastolic functions. The systolic-dicrotic notch pressure difference may serve as a discriminative marker between compensatory and noncompensatory tachycardia. In patients whose systolic-dicrotic notch pressure difference remained unchanged after esmolol infusion, stroke volume increased, and cardiac output remained steady as the heart rate dropped below 95 beats/min.^[39]^ Moreover, echocardiography and advanced hemodynamic monitoring could help evaluate the therapeutic effect of short-acting β-blockers in patients with septic shock, especially those with systolic dysfunction. Identifying patient subgroups that could benefit from ultrashort-acting β-blockers may represent a promising area for future research.

Landiolol is a highly selective β1-blocker associated with minimal negative inotropic and hypotensive effects. However, the recently published STRESS-L trial reported that landiolol did not reduce organ failure in patients with septic shock receiving norepinephrine.^[36]^ This suggests that the potential protective effects of β-blockers in sepsis may extend beyond their cardiac actions. Tan et al conducted a retrospective observational study involving septic patients from Europe and Australia/America (BEAST study) and reported that premorbid β-blockers, especially nonselective ones, might be associated with decreased mortality.^[43]^ Singer et al, in their retrospective study of Medicare beneficiaries admitted with sepsis, also reported a decrease in mortality associated with preadmission β-blockers, particularly nonselective β-blockers.^[11]^ Another retrospective study involving 1262 septic patients demonstrated that premorbid β1-selective blockers were associated with reduced norepinephrine usage and lower lactate concentrations within 6 hours after ICU admission. Notably, these patients also had a lower mortality rate. Patients receiving nonselective β-blockers also demonstrated a lower ICU mortality rate than nonusers, although this difference was not statistically significant (15.3% vs 20.6%).^[44]^ These findings have led to speculation that attenuation of the overactivated sympathetic nervous system and cytokine storm caused by sepsis may be critical factors. Nevertheless, given the retrospective nature of these studies, prospective RCTs are necessary to establish definitive causal relationships.

Sepsis is characterized by a cytokine storm, hypermetabolic state, and dysregulated immune function. Hyperinflammation typically manifests during the early phase of sepsis to eliminate pathogens. Elevated neutrophil-to-lymphocyte-to-platelet ratio, a marker of heightened inflammation response, has been associated with increased in-hospital mortality in COVID-19 patients.^[45]^ While, immunosuppression often develops in later stages of sepsis, which can impair pathogen clearance and increase susceptibility to opportunistic infections. The Prognostic Nutritional Index, calculated from total lymphocyte count and serum albumin, serves as an indicator of a patient’s immune status. A lower Prognostic Nutritional Index reflected severe inflammation and immune dysfunction, which has also been linked to higher mortality in critical ill patients.^[46,47]^ Beta-blockers may modulate immune dysfunction. For example, β1-blockers have been shown to downregulate proinflammatory responses, whereas β2-blockers may attenuate hypermetabolism and reduce proteolysis.^[4,48]^ Recent studies further suggest that β2-blockers could counteract norepinephrine-induced decreases in tumor necrosis factor-alpha and interleukin-6, while β1-blockers could not exert the same effect,^[49,50]^ although contrasting findings have also been reported.^[10,51]^ Notably, the immunological status of septic patients is heterogeneous and dynamic. Hyperinflammatory and immunosuppression often coexist during the early phase of sepsis.^[52]^ Consequently, the effects of β-blockers may vary across different immunological phenotypes. The impact of β-blockers on inflammation and immune function remains complex and sometimes contradictory, warranting further investigation into the underlying mechanisms.

## 5. Limitations

This study has several limitations that should be acknowledged. First, our analysis relied on articles retrieved from the WOS Core Collection database, which may have omitted certain relevant publications. Combining multiple databases through bibliometric software can pose challenges; however, it is worth noting that this database includes the most prominent medical journals. Second, our inclusion criteria were limited to articles written in English, potentially excluding those published in other languages. This language bias may have led to the omission of valuable research. Third, many articles, particularly those reporting RCTs, involve numerous authors and affiliated institutions. These factors could introduce variability into the results of bibliometric analysis. Fourth, some recently published articles may have received fewer citations despite their exceptional quality and substantial influence. This discrepancy may be inherent in bibliometric studies. Finally, it is essential to emphasize that our study primarily focused on bibliometric analysis and we did not undertake an exhaustive, in-depth mechanistic investigation.

## 6. Conclusion

There has been a discernible upward trend in the publication of articles related to β-blockers in sepsis, particularly in the last decade. Keyword publication burst detection revealed that the focus of this study shifted from excessive catecholamine release in the early phase of sepsis to the potential therapeutic role of β-blockers. The effects of β-blockers in sepsis and septic shock may depend on factors such as hemodynamic status, specific drug subtypes, and immunological phenotypes, suggesting these areas as potential focal points for future research. The United States leads in article publication, with substantial influence, while China exhibits a recent surge in publications, ranking second in publication strength. Complex international collaborations were evident, especially among those with numerous publications.

## Author contributions

**Conceptualization:** Xu-Ying Luo, Guangzhi Shi, Hong-Liang Li.

**Project administration:** Xu-Ying Luo, Hong-Liang Li.

**Software:** Xu-Ying Luo, Jie Zheng.

**Visualization:** Xu-Ying Luo, Jie Zheng.

**Writing – original draft:** Xu-Ying Luo, Jianfang Zhou, Jie Zheng.

**Writing – review & editing:** Xu-Ying Luo, Jianfang Zhou, Guangzhi Shi, Hong-Liang Li.

**Data curation:** Jianfang Zhou.

**Methodology:** Jie Zheng, Hong-Liang Li.

**Supervision:** Guangzhi Shi, Hong-Liang Li.

**Funding acquisition:** Hong-Liang Li.

## Supplementary Material
