# Supplementary material for: The global trends and hotspots beta-blocker therapy in sepsis and septic shock: A bibliometric analysis based on CiteSpace and VOSviewer
Source: Medicine (Baltimore). 2026 Apr 24;105(17):e48347. doi: 10.1097/MD.0000000000048347 (PMC13124423; doi:10.1097/MD.0000000000048347)
Supplement: Supplementary file 1 [file medi-105-e48347-s001.pdf]

The global trends and hotspots beta-blocker therapy in sepsis and septic shock: A bibliometric analysis based on CiteSpace and VOSviewer

Xu- Ying Luo, MD, Jianfang Zhou, MD, Jie Zheng, MSc, Guangzhi Shi, MD, Hong- Liang Li, MD*

Table S1. Top 10 most cited references

Rank	Title	Author	Journal	Type	Year	IF (2022)	Centrality	Cluster ID
1	Effect of heart rate control with esmolol on hemodynamic and clinical outcomes in patients with septic shock: a randomized clinical trial	Morelli A, et al	JAMA	Article	2014	120.7	0.04	2
2	The Third International Consensus Definitions for Sepsis and Septic Shock (Sepsis-3)	Singer M, et al	JAMA	Article	2016	120.7	0.12	4
3	Microvascular effects of heart rate control with esmolol in patients with septic shock: a pilot study	Morelli A, et al	Crit Care Med	Article	2013	9.3	0.03	2
4	Heart rate reduction with esmolol is associated with improved arterial elastance in patients with septic shock: a prospective observational study	Morelli A, et al	Intensive Care Med	Article	2016	41.8	0.01	2
5	Cardioprotection, attenuated systemic inflammation, and survival benefit of beta1-adrenoceptor blockade in severe sepsis in rats	Ackland GL, et al	Crit Care Med	Article	2010	9.3	0.06	0
6	Surviving Sepsis Campaign: international guidelines for management of severe sepsis and septic shock, 2012	Dellinger RP, et al	Intensive Care Med	Guideline	2013	41.8	0	2
7	β1-Adrenergic Inhibition Improves Cardiac and Vascular Function in Experimental Septic Shock	Kimmoun A, et al	Crit Care Med	Article	2015	9.3	0.02	2
8	Effects of esmolol on systemic and pulmonary hemodynamics and on oxygenation in pigs with hypodynamic endotoxin shock	Aboab J, et al	Intensive Care Med	Article	2011	41.8	0.03	0
9	Beta-blocker use in severe sepsis and septic shock: a systematic review	Sanfilippo F, et al	Curr Med Res Opin	Review	2015	2.71	0	2
10	Continuing chronic beta-blockade in the acute phase of severe sepsis and septic shock is associated with decreased mortality rates up to 90 days	Fuchs C, et al	Br J Anaesth	Article	2017	11.7	0.05	1
