# Supplementary figures and images for: The global trends and hotspots beta-blocker therapy in sepsis and septic shock: A bibliometric analysis based on CiteSpace and VOSviewer
Source: Medicine (Baltimore). 2026 Apr 24;105(17):e48347. doi: 10.1097/MD.0000000000048347 (PMC13124423; doi:10.1097/MD.0000000000048347)

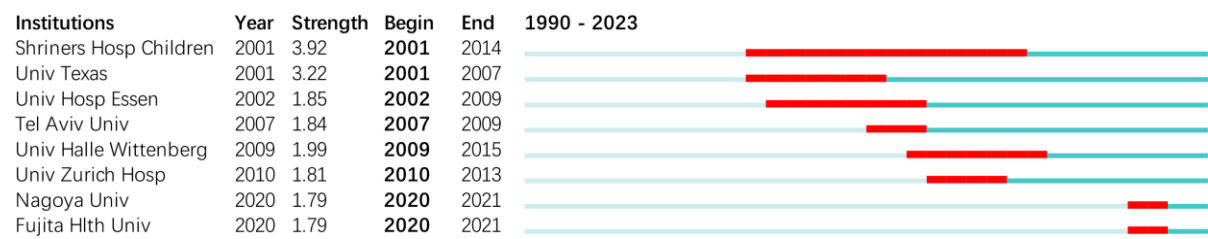

Supplementary Figure S1. Publication bursts of institutions sorted by start time

Supplement: Supplementary file 2 [file medi-105-e48347-s002.pdf]
